# Poly[aqua­hemi(μ_4_-oxalato)[μ_3_-5-(pyrazin-2-yl)tetra­zolato]cadmium(II)]

**DOI:** 10.1107/S1600536810027406

**Published:** 2010-07-17

**Authors:** Chen Zhang, Ting-Ting Wang

**Affiliations:** a School of Chemistry and Environment, South China Normal University, Guangzhou 510006, People’s Republic of China; b School of Chemistry and Chemical Engineering, South China University of Technology, Guangzhou 510640, People’s Republic of China

## Abstract

In the title polymeric complex, [Cd(C_5_H_3_N_6_)(C_2_O_4_)_0.5_(H_2_O)]_*n*_, the Cd^II^ ion is coordinated by four O atoms and three N atoms from two 5-(pyrazin-2-yl)tetra­zolate ligands, two oxalate ligands and one water mol­ecule, displaying a distorted monocapped octa­hedral geometry. The bridging ligands link metal centres, forming a three-dimensional network which is stabilized by inter­molecular O—H⋯N hydrogen-bonding inter­actions.

## Related literature

For related structures, see: Deng *et al.* (2007 ▶); Zeng *et al.* (2007 ▶). For graph-set notation, see: Bernstein *et al.* (1995 ▶).
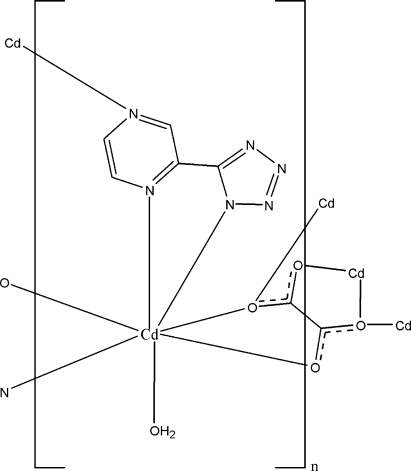


## Experimental

### 

#### Crystal data

[Cd(C_5_H_3_N_6_)(C_2_O_4_)_0.5_(H_2_O)]
*M* *_r_* = 321.56
Monoclinic, 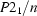

*a* = 5.8801 (1) Å
*b* = 13.1286 (2) Å
*c* = 11.5647 (2) Å
β = 94.867 (1)°
*V* = 889.55 (3) Å^3^
*Z* = 4
Mo *K*α radiation
μ = 2.46 mm^−1^
*T* = 296 K
0.24 × 0.22 × 0.19 mm

#### Data collection

Bruker APEXII area-detector diffractometer
Absorption correction: multi-scan (*SADABS*; Sheldrick, 2008*a* ▶) *T*_min_ = 0.590, *T*_max_ = 0.652
7467 measured reflections
1588 independent reflections
1566 reflections with *I* > 2σ(*I*)
*R*_int_ = 0.031

#### Refinement

*R*[*F* ^2^ > 2σ(*F* ^2^)] = 0.023
*wR*(*F* ^2^) = 0.056
*S* = 1.19
1588 reflections
151 parameters
3 restraints
H atoms treated by a mixture of independent and constrained refinement
Δρ_max_ = 0.33 e Å^−3^
Δρ_min_ = −0.77 e Å^−3^

### 

Data collection: *APEX2* (Bruker, 2004 ▶); cell refinement: *SAINT* (Bruker, 2004 ▶); data reduction: *SAINT*; program(s) used to solve structure: *SHELXS97* (Sheldrick, 2008*b* ▶); program(s) used to refine structure: *SHELXL97* (Sheldrick, 2008*b* ▶); molecular graphics: *SHELXTL* (Sheldrick, 2008*b* ▶); software used to prepare material for publication: *SHELXTL*.

## Supplementary Material

Crystal structure: contains datablocks I, global. DOI: 10.1107/S1600536810027406/rz2474sup1.cif

Structure factors: contains datablocks I. DOI: 10.1107/S1600536810027406/rz2474Isup2.hkl

Additional supplementary materials:  crystallographic information; 3D view; checkCIF report

## Figures and Tables

**Table 1:** Hydrogen-bond geometry (Å, °)

*D*—H⋯*A*	*D*—H	H⋯*A*	*D*⋯*A*	*D*—H⋯*A*
O1*W*—H2*W*⋯N4^i^	0.82 (3)	2.08 (3)	2.897 (4)	174 (4)
O1*W*—H1*W*⋯N3^ii^	0.82 (3)	1.93 (3)	2.757 (4)	179 (5)

Symmetry codes: (i) 
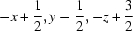
; (ii) 
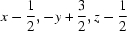
.

**Fig. 1.**
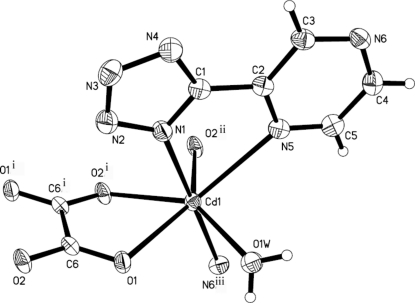
The molecular structure of the title complound showing the atomic-numbering scheme and displacement ellipsoids drawn at the 50% probability level. Symmetry codes: (i) 1-x, 1-y, 2-z; (ii) -1+x, y, z; (iii) -0.5-x, -1/2+y, 1.5-z.

**Fig. 2.**
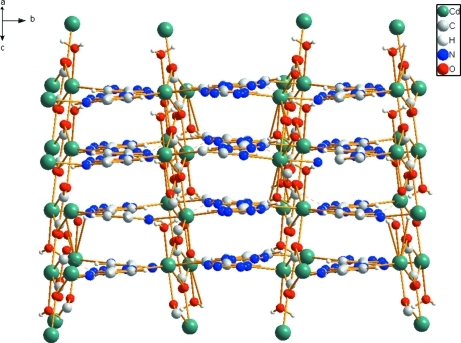
A view of the three-dimensional network of the title compound. Hydrogen bonds are shown as dashed lines.

## supplementary crystallographic information

### Comment

In recent years, research on coordination polymers has made considerable progress in the fields of supramolecular chemistry and crystal engineering, because of their intriguing structural motifs and functional properties, such as molecular adsorption, magnetism, and luminescence. The reports on tetrazoles are expanding rapidly, since tetrazoles have an important role in coordination chemistry as ligands (Deng *et al.* 2007; Zeng *et al.* 2007). In the general reaction, tetrazoles are prepared by the addition of an azide to nitriles in water with the aid of a Lewis acid such a Zn^2+^. In this paper is reported the crystal structure of the title coordination polymer, which has been obtained under hydrothermal condition using 2-cyanopyrazine, NaN_3_, oxalic acid and the Lewis acid CdCl_2_ as reagents.

In the structure of the title compound (Fig. 1), each cadmium(II) centre is seven-coordinated by four O atoms and three N atoms from two 5-(2-pyrazinyl)tetrazolate ligands, two oxalate ligands and one water molecule, and can described as having a distorted monocapped octahedral geometry with Cd···O and Cd···N distances ranging from 2.312 (2) to 2.404 (2) Å and from 2.284 (3) to 2.700 (3) Å, respectively. The 5-(2-pyrazinyl)tetrazolate and oxalate ligands act as bridging ligands, linking the metal centres to assemble a three-dimensional motif (Fig. 2). Within the three-dimensional network, centrosymmetrically related water molecules interact with adjacent tetrazolate ligands through O—H···N hydrogen bonds to form ten-membered rings with *R*_4_^4^(10) motifs (Bernstein *et al.*, 1995).

### Experimental

A mixture of CdCl_2_ (0.183 g; 1 mmol), 2-cyanopyrazine (0.105 g; 1 mmol), oxalic acid (0.09 g; 1 mmol) and NaN_3_ (0.065, 1 mmol) in water (10 ml) was stirred vigorously for 30 min and then sealed in a Teflon-lined stainless-steel autoclave (20 ml capacity). The autoclave was heated and maintained at 422 K for 50 h, and then cooled to room temperature at 5 K h^-1^. Colourless block crystals suitable for X-ray analysis were obtained.

### Refinement

Water H atoms were located in a difference Fourier map and were refined with distance restraints of O–H = 0.82 Å and H···H = 1.35 Å, and with *U*_iso_(H) = 1.5 *U*_eq_(O). Other H atoms were placed in calculated positions (C—H = 0.93 Å) and refined using a riding model, with *U*_iso_(H) = 1.2*U*_eq_(C)

### Crystal data

**Table:**

[Cd(C_5_H_3_N_6_)(C_2_O_4_)_0.5_(H_2_O)]	*F*(000) = 620
*M**_r_* = 321.56	*D*_x_ = 2.401 Mg m^−^^3^
Monoclinic, *P*2_1_/*n*	Mo *K*α radiation, λ = 0.71073 Å
Hall symbol: -P 2yn	Cell parameters from 1076 reflections
*a* = 5.8801 (1) Å	θ = 1.4–28.0°
*b* = 13.1286 (2) Å	µ = 2.46 mm^−^^1^
*c* = 11.5647 (2) Å	*T* = 296 K
β = 94.867 (1)°	Block, colourless
*V* = 889.55 (3) Å^3^	0.24 × 0.22 × 0.19 mm
*Z* = 4	

### Data collection

**Table:**

Bruker APEXII area-detector diffractometer	1588 independent reflections
Radiation source: fine-focus sealed tube	1566 reflections with *I* > 2σ(*I*)
graphite	*R*_int_ = 0.031
φ and ω scan	θ_max_ = 25.2°, θ_min_ = 2.4°
Absorption correction: multi-scan (*SADABS*; Sheldrick, 2008a)	*h* = −7→7
*T*_min_ = 0.590, *T*_max_ = 0.652	*k* = −13→15
7467 measured reflections	*l* = −12→13

### Refinement

**Table:**

Refinement on *F*^2^	Primary atom site location: structure-invariant direct methods
Least-squares matrix: full	Secondary atom site location: difference Fourier map
*R*[*F*^2^ > 2σ(*F*^2^)] = 0.023	Hydrogen site location: inferred from neighbouring sites
*wR*(*F*^2^) = 0.056	H atoms treated by a mixture of independent and constrained refinement
*S* = 1.19	*w* = 1/[σ^2^(*F*_o_^2^) + (0.0221*P*)^2^ + 1.3797*P*] where *P* = (*F*_o_^2^ + 2*F*_c_^2^)/3
1588 reflections	(Δ/σ)_max_ = 0.001
151 parameters	Δρ_max_ = 0.33 e Å^−^^3^
3 restraints	Δρ_min_ = −0.77 e Å^−^^3^

### Special details

**Table:**

Geometry. All e.s.d.'s (except the e.s.d. in the dihedral angle between two l.s. planes) are estimated using the full covariance matrix. The cell e.s.d.'s are taken into account individually in the estimation of e.s.d.'s in distances, angles and torsion angles; correlations between e.s.d.'s in cell parameters are only used when they are defined by crystal symmetry. An approximate (isotropic) treatment of cell e.s.d.'s is used for estimating e.s.d.'s involving l.s. planes.
Refinement. Refinement of *F*^2^ against ALL reflections. The weighted *R*-factor *wR* and goodness of fit *S* are based on *F*^2^, conventional *R*-factors *R* are based on *F*, with *F* set to zero for negative *F*^2^. The threshold expression of *F*^2^ > σ(*F*^2^) is used only for calculating *R*-factors(gt) *etc*. and is not relevant to the choice of reflections for refinement. *R*-factors based on *F*^2^ are statistically about twice as large as those based on *F*, and *R*- factors based on ALL data will be even larger.

### Fractional atomic coordinates and isotropic or equivalent isotropic displacement parameters (Å^2^)

**Table:**

	*x*	*y*	*z*	*U*_iso_*/*U*_eq_	
Cd1	0.06773 (3)	0.544930 (16)	0.847068 (19)	0.01725 (10)	
C1	0.0857 (5)	0.7869 (2)	0.8871 (3)	0.0205 (6)	
C2	−0.1305 (5)	0.7892 (2)	0.8144 (3)	0.0199 (6)	
C3	−0.2443 (6)	0.8798 (2)	0.7882 (3)	0.0246 (7)	
H3	−0.1854	0.9402	0.8203	0.029*	
C4	−0.5172 (6)	0.7932 (3)	0.6778 (3)	0.0269 (7)	
H4	−0.6510	0.7919	0.6288	0.032*	
C5	−0.4074 (6)	0.7024 (3)	0.7069 (3)	0.0250 (7)	
H5	−0.4724	0.6416	0.6793	0.030*	
C6	0.5686 (5)	0.5102 (2)	0.9467 (3)	0.0180 (6)	
N1	0.1910 (5)	0.6999 (2)	0.9168 (2)	0.0227 (6)	
N2	0.3821 (5)	0.7263 (2)	0.9813 (3)	0.0287 (7)	
N3	0.3872 (5)	0.8258 (2)	0.9887 (3)	0.0287 (6)	
N4	0.2027 (5)	0.8672 (2)	0.9305 (3)	0.0270 (6)	
N5	−0.2106 (5)	0.69963 (19)	0.7735 (2)	0.0220 (6)	
N6	−0.4366 (5)	0.8823 (2)	0.7180 (2)	0.0232 (6)	
O1	0.4638 (4)	0.51192 (18)	0.84910 (19)	0.0227 (5)	
O2	0.7803 (4)	0.52289 (17)	0.9693 (2)	0.0225 (5)	
O1W	0.1382 (5)	0.56000 (19)	0.6539 (2)	0.0317 (6)	
H1W	0.062 (7)	0.594 (2)	0.605 (3)	0.048*	
H2W	0.176 (7)	0.5058 (18)	0.626 (3)	0.048*	

### Atomic displacement parameters (Å^2^)

**Table:**

	*U*^11^	*U*^22^	*U*^33^	*U*^12^	*U*^13^	*U*^23^
Cd1	0.01395 (14)	0.01826 (15)	0.01932 (15)	−0.00172 (8)	0.00015 (9)	−0.00031 (8)
C1	0.0230 (16)	0.0187 (15)	0.0206 (16)	−0.0008 (12)	0.0063 (12)	0.0033 (13)
C2	0.0198 (15)	0.0194 (15)	0.0214 (16)	−0.0016 (12)	0.0067 (12)	0.0020 (13)
C3	0.0279 (18)	0.0195 (16)	0.0268 (18)	−0.0002 (13)	0.0052 (14)	0.0011 (13)
C4	0.0233 (16)	0.0327 (19)	0.0247 (17)	−0.0013 (14)	0.0020 (13)	0.0040 (15)
C5	0.0245 (17)	0.0231 (16)	0.0279 (18)	−0.0063 (13)	0.0051 (14)	0.0011 (14)
C6	0.0140 (15)	0.0176 (14)	0.0224 (16)	0.0027 (12)	0.0022 (12)	0.0030 (13)
N1	0.0203 (13)	0.0204 (13)	0.0269 (15)	0.0005 (11)	−0.0007 (11)	−0.0007 (11)
N2	0.0237 (15)	0.0298 (16)	0.0319 (16)	−0.0002 (12)	−0.0017 (12)	−0.0028 (13)
N3	0.0304 (16)	0.0283 (15)	0.0270 (16)	−0.0070 (12)	−0.0008 (12)	−0.0015 (12)
N4	0.0296 (15)	0.0201 (14)	0.0306 (16)	−0.0028 (12)	−0.0022 (12)	0.0012 (12)
N5	0.0241 (14)	0.0200 (14)	0.0226 (14)	−0.0005 (11)	0.0063 (11)	0.0028 (11)
N6	0.0221 (14)	0.0233 (14)	0.0245 (14)	0.0038 (11)	0.0042 (11)	0.0025 (12)
O1	0.0159 (11)	0.0330 (12)	0.0190 (12)	0.0023 (9)	−0.0005 (9)	0.0025 (10)
O2	0.0116 (11)	0.0316 (12)	0.0242 (12)	0.0016 (9)	0.0016 (9)	0.0070 (10)
O1W	0.0432 (16)	0.0285 (13)	0.0229 (13)	0.0128 (11)	−0.0003 (11)	0.0006 (10)

### Geometric parameters (Å, °)

**Table:**

Cd1—N1	2.284 (3)	C4—C5	1.383 (5)
Cd1—O2^i^	2.312 (2)	C4—H4	0.9300
Cd1—O1W	2.315 (3)	C5—N5	1.335 (4)
Cd1—O1	2.367 (2)	C5—H5	0.9300
Cd1—N5	2.700 (3)	C6—O1	1.239 (4)
Cd1—N6^ii^	2.371 (3)	C6—O2	1.261 (4)
Cd1—O2^iii^	2.404 (2)	C6—C6^iii^	1.553 (6)
C1—N1	1.330 (4)	N1—N2	1.341 (4)
C1—N4	1.333 (4)	N2—N3	1.309 (4)
C1—C2	1.464 (4)	N3—N4	1.342 (4)
C2—N5	1.338 (4)	N6—Cd1^iv^	2.371 (3)
C2—C3	1.385 (4)	O2—Cd1^v^	2.312 (2)
C3—N6	1.335 (4)	O2—Cd1^iii^	2.404 (2)
C3—H3	0.9300	O1W—H1W	0.82 (3)
C4—N6	1.332 (5)	O1W—H2W	0.82 (3)
			
N1—Cd1—O2^i^	97.03 (9)	N6—C4—H4	119.1
N1—Cd1—O1W	100.78 (10)	C5—C4—H4	119.1
O2^i^—Cd1—O1W	143.20 (9)	N5—C5—C4	121.9 (3)
N1—Cd1—O1	82.94 (9)	N5—C5—H5	119.0
O2^i^—Cd1—O1	137.80 (8)	C4—C5—H5	119.0
O1W—Cd1—O1	76.63 (9)	O1—C6—O2	126.3 (3)
N1—Cd1—N6^ii^	177.81 (10)	O1—C6—C6^iii^	118.4 (3)
O2^i^—Cd1—N6^ii^	81.17 (9)	O2—C6—C6^iii^	115.3 (3)
O1W—Cd1—N6^ii^	81.40 (9)	C1—N1—N2	105.8 (3)
O1—Cd1—N6^ii^	97.56 (9)	C1—N1—Cd1	123.2 (2)
N1—Cd1—O2^iii^	86.28 (9)	N2—N1—Cd1	130.7 (2)
O2^i^—Cd1—O2^iii^	69.50 (8)	N3—N2—N1	107.9 (3)
O1W—Cd1—O2^iii^	143.19 (8)	N2—N3—N4	111.0 (3)
O1—Cd1—O2^iii^	68.39 (7)	C1—N4—N3	103.8 (3)
N6^ii^—Cd1—O2^iii^	91.92 (9)	C5—N5—C2	116.2 (3)
N1—C1—N4	111.6 (3)	C4—N6—C3	116.7 (3)
N1—C1—C2	121.9 (3)	C4—N6—Cd1^iv^	125.7 (2)
N4—C1—C2	126.5 (3)	C3—N6—Cd1^iv^	116.8 (2)
N5—C2—C3	121.9 (3)	C6—O1—Cd1	115.17 (19)
N5—C2—C1	116.6 (3)	C6—O2—Cd1^v^	130.5 (2)
C3—C2—C1	121.5 (3)	C6—O2—Cd1^iii^	114.90 (19)
N6—C3—C2	121.5 (3)	Cd1^v^—O2—Cd1^iii^	110.50 (8)
N6—C3—H3	119.3	Cd1—O1W—H1W	126 (3)
C2—C3—H3	119.3	Cd1—O1W—H2W	112 (3)
N6—C4—C5	121.8 (3)	H1W—O1W—H2W	110.4 (18)

Symmetry codes: (i) *x*−1, *y*, *z*; (ii) −*x*−1/2, *y*−1/2, −*z*+3/2; (iii) −*x*+1, −*y*+1, −*z*+2; (iv) −*x*−1/2, *y*+1/2, −*z*+3/2; (v) *x*+1, *y*, *z*.

### Hydrogen-bond geometry (Å, °)

**Table:**

*D*—H···*A*	*D*—H	H···*A*	*D*···*A*	*D*—H···*A*
O1W—H2W···N4^vi^	0.82 (3)	2.08 (3)	2.897 (4)	174 (4)
O1W—H1W···N3^vii^	0.82 (3)	1.93 (3)	2.757 (4)	179 (5)

Symmetry codes: (vi) −*x*+1/2, *y*−1/2, −*z*+3/2; (vii) *x*−1/2, −*y*+3/2, *z*−1/2.
